# Ideal Test Time for Coronavirus Disease 2019 Contact Tracing

**DOI:** 10.3389/fpubh.2021.690006

**Published:** 2022-01-28

**Authors:** Shigeta Miyake, Hideaki Kato, Nobuko Tanaka, Kohei Shimizu, Hiroki Ozawa, Chiharu Kawakami, Shuzo Usuku, Hideaki Nakajima, Tetsuya Yamamoto

**Affiliations:** ^1^Infection Prevention and Control Department, Yokohama City University Hospital, Yokohama, Japan; ^2^Department of Neurosurgery, Yokohama City University Graduate School of Medicine, Yokohama, Japan; ^3^Department of Stem Cell and Immune Regulation, Yokohama City University Graduate School of Medicine, Yokohama, Japan; ^4^Yokohama City Institute of Public Health, Yokohama, Japan

**Keywords:** coronavirus disease 2019, contact duration, contact tracing, cycle threshold, risk factor, household contact

## Abstract

**Background:**

Epidemiological contact tracing is a powerful tool to rapidly detect SARS-CoV-2 infection in persons with a close contact history with COVID-19-affected patients. However, it remains unclear whom and when should be PCR tested among the close contact subjects.

**Methods:**

We retrospectively analyzed 817 close contact subjects, including 144 potentially SARS-CoV-2-infected persons. The patient characteristics and contact type, duration between the date of the close contact and specimen sampling, and PCR test results in PCR positive and negative persons were compared.

**Results:**

We found that male gender {adjusted odds ratio 1.747 [95% confidence interval (CI) 1.180–2.608]}, age ≥ 60 [1.749 (95% CI 1.07–2.812)], and household contact [2.14 (95% CI 1.388–3.371)] are independent risk factors for close contact SARS-CoV-2 infection. Symptomatic subjects were predicted 6.179 (95% CI 3.985–9.61) times more likely to be infected compared to asymptomatic ones. We could observe PCR test positivity between days 1 and 17 after close contact. However, no subject could be found with a Ct-value <30, considered less infective, after day 14 of close contact.

**Conclusions:**

Based on our results, we suggest that contact tracing should be performed on the high-risk subjects between days 3 and 13 after close contacts.

## Introduction

The ongoing coronavirus disease (COVID-19) pandemic is one of the biggest global challenges for the healthcare and economic systems (1). COVID-19, caused by severe acute respiratory syndrome coronavirus 2 (SARS-CoV-2), has affected more than 150 million individuals and caused over 2.3 million deaths as of February 21, 2021 (2). The case fatality rates of COVID-19 differ from country to country, depending on the healthcare systems and health policies (3). Exceeding the healthcare capacity of intensive care units could lead to the collapse of the medical services and trigger a mortality rate increase (4, 5). During the pandemic, several aspects of the characteristics of SARS-CoV-2 have been unveiled (6), indicating that asymptomatic and pre-symptomatic carriers are potentially important infection sources. To control the spread of infection, it is important to intervene in the transmission chain. Adequate preventive measures, such as social distancing, hand hygiene, and wearing masks, are recommended for the public (7). At the national level, it is necessary to apply quarantine policies for infected and suspected cases, including asymptomatic ones (8, 9).

COVID-19 affected 0.4 million individuals and caused 6.4 thousand deaths in Japan as of February 9, 2021 (10). Close contacts identified using the Japanese guideline bore a high infection probability (11). Yokohama is the second largest city in Japan, and the number of COVID-19 cases was ~18,800 (0.5% of the population) as of February 9, 2021 (12). Three different COVID-19 waves occurred in Japan: April 2020, July to August 2020, and January 2021. The number of infected persons was relatively low compared to that in other countries. A potential reason for the low infection numbers could be thanked to active epidemiological surveys and contact tracing, which turned prove to be effective virus control policies in Japan (13). The public health centers of local governments conducted active epidemiological surveys for contact tracing driven by the intention to minimize cluster outbreaks and limit spread of the infection. These surveys enable the early detection and isolation of asymptomatic COVID-19 patients. Since February 2020, Yokohama City University Hospital has provided an outpatient clinic for contact tracing, in collaboration with the local government. As the COVID-19 transmission dynamics in the close contact of infected individuals are not understood well-enough, the outpatient clinic for contact tracing has been targeting this particular population. However, the risk factors for SARS-CoV-2 infection in the close contact cohort are still unknown. Moreover, it also remains elusive when to screen the close contact persons. In this study, we investigated the risk factors for SARS-CoV-2 infection between close contact persons and COVID-19 patients and established the ideal timeframe of sample collection for screening.

## Materials and Methods

This single-center retrospective cohort study aimed at optimizing epidemiological COVID-19 contact tracing surveys at Yokohama City University Hospital from February 1, 2020, to January 31, 2021. All patients who visited our outpatient clinic for COVID-19 contact tracing during the study period were included in the investigation. The close contacts of patients with COVID-19 were identified by the public health center in Japan according to the official criteria: contact with <1 m distance, contact >15 min, and contact without wearing adequate masks with or without symptoms. This policy was maintained throughout this study period. When the public center noticed the close contact cases regarding to the criteria, the public center performed a contact tracing investigation and PCR test for all subjects who met the official criteria as soon as possible. All close contacts were randomly allocated to a specialized medical institution for investigation by the public health center. The exclusion criteria included overseas travelers and those with repeated visits for negative PCR confirmation. There is no repetitive test per person included in the analysis. After excluding 105 subjects, finally 817 subjects were retrospectively analyzed. The following patient characteristics were collected: age, sex, contact type (household, verbal interactions such as meetings at workplaces, eating a meal together, and other types of close contact), duration between the date of the close contact and specimen sampling, and PCR test results. When the contact was continuous, the duration of contact was calculated starting from the onset date of the patient in contact. We also analyzed whether the patient was symptomatic or asymptomatic. The symptoms included fever, respiratory symptoms, digestive symptoms, and loss of smell or taste. Patient data were retrospectively examined using medical records. The Institutional Review Board of Yokohama City University Hospital approved this study (approval number B200200047). For all patients, consent for participation for this retrospective study was obtained by disclosing the clinical study, including the description of opt-out (https://www.yokohama-cu.ac.jp/amedrc/ethics/ethical/fuzoku_optout.html).

### Outpatient Clinic for Contact Tracing

Patients referring to our hospital were placed in a separate outpatient clinic in the emergency room. After the clinical interview, nasopharyngeal swab or saliva samples were collected for PCR testing using a nasopharyngeal swab and transport media (COPAN, Brescia, Italia) or 2 mL of saliva. Saliva sampling specimens were preferred after their approval for PCR testing in June 2020. A nasopharyngeal swab was used for testing subjects before June 2020 and those who ate or drink within 30 min of sampling specimens. The applied collection method was chosen individually after consultation with the patient. The collected PCR samples were packed securely and sent to the Yokohama City Institute of Public Health for PCR testing. The PCR testing was performed according to the Manual for Detection of Pathogen 2019-nCoV provided by the National Institute of Infectious Disease in Japan (14).

### Statistical Analysis

The results are presented as the mean for the quantitative data and frequency (percentage) for the categorical data. Continuous data are presented as means and 95% confidence intervals (CIs) or medians and interquartile ranges (IQRs). Data were analyzed by performing two-tailed Mann–Whitney *U*-test for comparisons of continuous variables between two groups and Fisher's exact test for comparisons of categorical data. Multivariate logistic regression analyses were performed to investigate the predictors of SARS-CoV-2 positivity, and adjustments were made for potential confounders: male sex, age (including age ≥ 60 years), presence of symptoms, sampling specimens, and type of contact. The results of bivariate analysis indicated that the listed factors contributed significantly to SARS-CoV-2 positivity. Simple linear regression analysis was used for analyzing the association between the date after the contact and the Ct values of PCR specimens. *P* < 0.05 were considered statistically significant. All statistical analyses were performed using the JMP Pro 15 (SAS Institute Inc., Cary, NC, USA) and Prism 7.9 J softwares for Windows and the Prism 9.0 software for Macintosh (GraphPad Software, Inc., San Diego, CA, USA).

## Results

### Outpatient Characteristics for Contact Tracing

Between February 1, 2020, and January 31, 2021, a total of 922 outpatients allocated by the public health center underwent a medical examination at our hospital. Of these outpatients, 105 were excluded due to our exclusion criteria. Hence, a total of 817 consecutive patients were enrolled in this study. The mean participant age was 36.2 years (range: 0–91 years), and 409 patients (50.1%) were men. The mean duration between the contact and sample collection was 7.0 days (range: 0–19 days). All subjects were asymptomatic or had very mild symptoms at the time of visit. A total of 701 (85.8%) patients were asymptomatic and 505 (61.8%) patients were assessed using nasopharyngeal swab samples. The most common contact type was household contact (523, 64.0%), followed by eating together (142, 17.4%), talking (109, 13.3%), and others (43, 5.3%) (Table 1).

**Table 1:** Screened characteristics of the subjects in epidemiological close contact tracing.

		***N* (%)**	**PCR positive**	**PCR negative**	***P*-value**
Gender	male	409 (50.1)	83 (57.6)	326 (48.4)	0.0536
	female	408 (49.9)	61 (42.4)	347 (51.6)	
Age	Age ≥ 60	137 (16.8)	33 (22.9)	104 (15.5)	0.0363^*^
	Age <60	680 (83.2)	111 (77.1)	569 (84.5)	
Subject symptoms	Symptomatic	116 (14.2)	54 (37.5)	62 (9.2)	<0.0001^*^
	Asymptomatic	701 (85.8)	90 (62.5)	611 (90.8)	
Specimen sampling	Nasopharyngeal swab	505 (61.8)	96 (66.7)	409 (60.8)	0.1874
	Saliva	312 (38.2)	48 (33.3)	265 (39.4)	
Types of contacts	Household contact	523 (64.0)	109 (75.7)	414 (61.5)	0.0011^*^^†^
	Eat together	142 (17.4)	16 (11.1)	126 (18.7)	
	Talk together	109 (13.3)	14 (9.7)	95 (14.1)	
	Other	43 (5.3)	5 (3.5)	38 (5.6)	

* *Statistically significant by Fisher's exact test*.

† *Compared with eat together, talk together, and others*.

### PCR Positivity

Overall, 144 (17.6%) patients tested positive for SARS-CoV-2 by PCR. In total, 19.0% (96/505) and 15.4% (48/312) of the patients tested positive for SARS-CoV-2 by PCR using a nasopharyngeal swab and saliva samples, respectively. Our univariate analysis showed that the number of household contact subjects (75.7%) was significantly higher in the PCR positive group compared with the PCR negative group. Moreover, the numbers of subjects with symptoms (37.5 vs. 9.2%) and subjects aged ≥60 years (22.9 vs. 15.5%) were higher in the PCR positive group compared with PCR negative group (Table 1). Our multivariate analysis, performed to exclude confounding biases, showed that male gender (adjusted odds ratio, 1.747), age ≥60 (1.749), being symptomatic at hospital visit (6.179), and household contact (2.14) were risk factors for SARS-CoV-2 infection for the close contact subjects with COVID-19 (Table 2).

**Table 2:** Multivariate analysis of risk factors for SARS-CoV-2 infection on the close contact persons of patients with COVID-19.

	**Adjusted odds ratio [95% confidence interval]**
Male gender	1.747 [1.18, 2.608]^*^
Age ≥ 60 year old	1.749 [1.07, 2.812]^*^
Symptomatic	6.179 [3.985, 9.61]^*^
Nasopharyngeal swab^†^	1.226 [0.8184, 1.855]
Household contact^‡^	2.14 [1.388, 3.371]^*^

* *Statistically significant*.

† *Compared with saliva specimens*.

‡ *Compared with eat together, talk together, and others*.

### PCR Positivity and Duration Between Close Contact and Specimen Collection

**Figure 1:**
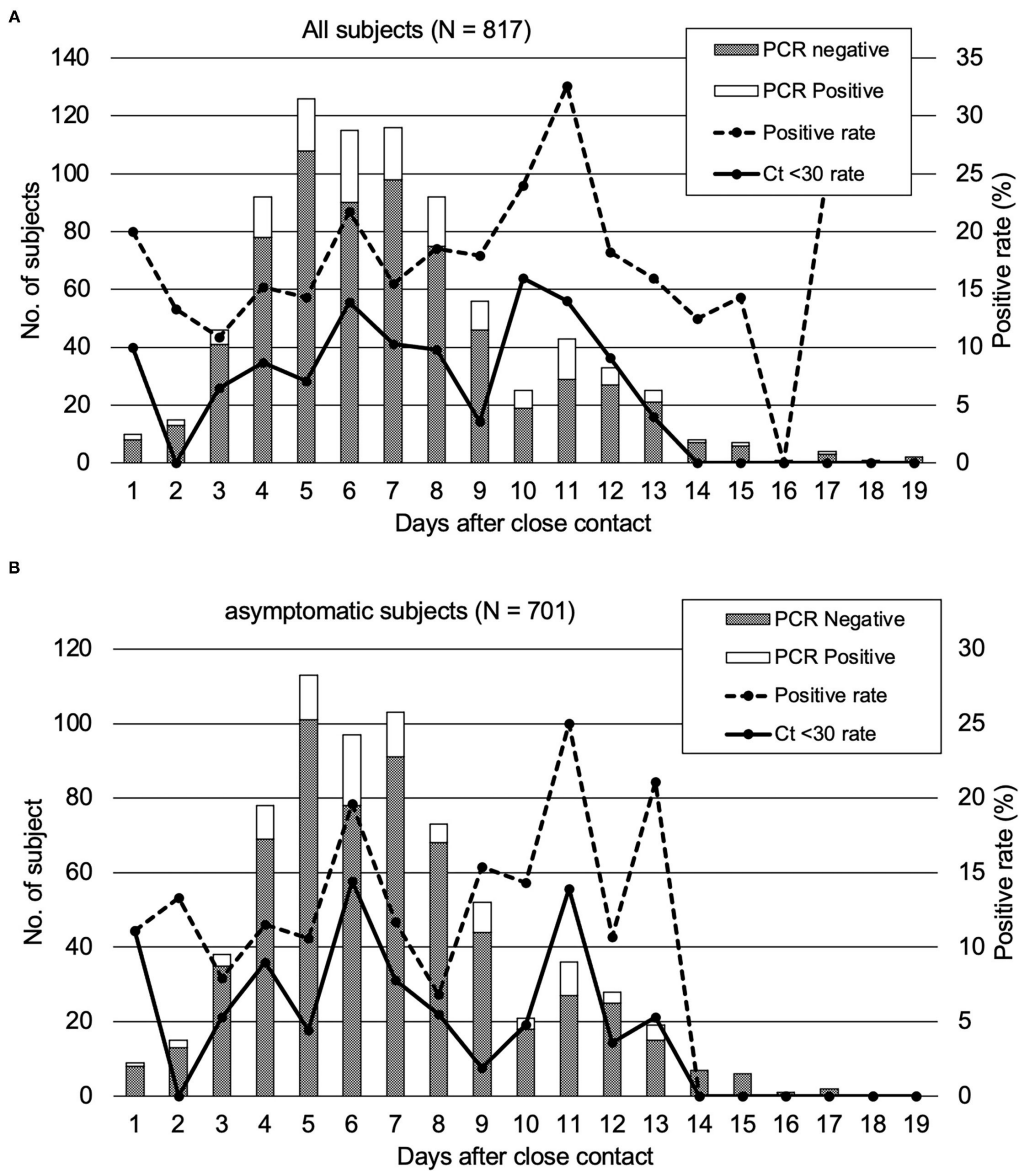
PCR positivity of the subjects who underwent close contact PCR tests. The rates of the subjects with a high viral load (Ct < 30) were also plotted. **(A)** All close contact subjects in our study. **(B)** asymptomatic subjects.

We analyzed the duration after close contact to specimen collection and PCR positivity of the subjects having close contact with COVID-19. Of the 817 subjects, the PCR positivity rates were the highest on day 11 (32.6%) and were higher than 10% between days 3 and 15 (Figure 1A). Of 701 asymptomatic subjects at the time of specimen collection, the PCR positivity rate was over 10% between days 4 and 13, and the highest rate of PCR positivity could be observed on day 11 (25.0%) (Figure 1B). There were no subjects with Ct values <30 observed on days 14–17.

### Association Between Duration of Specimen Collection and Ct Values of PCR Positive Subjects

**Figure 2:**
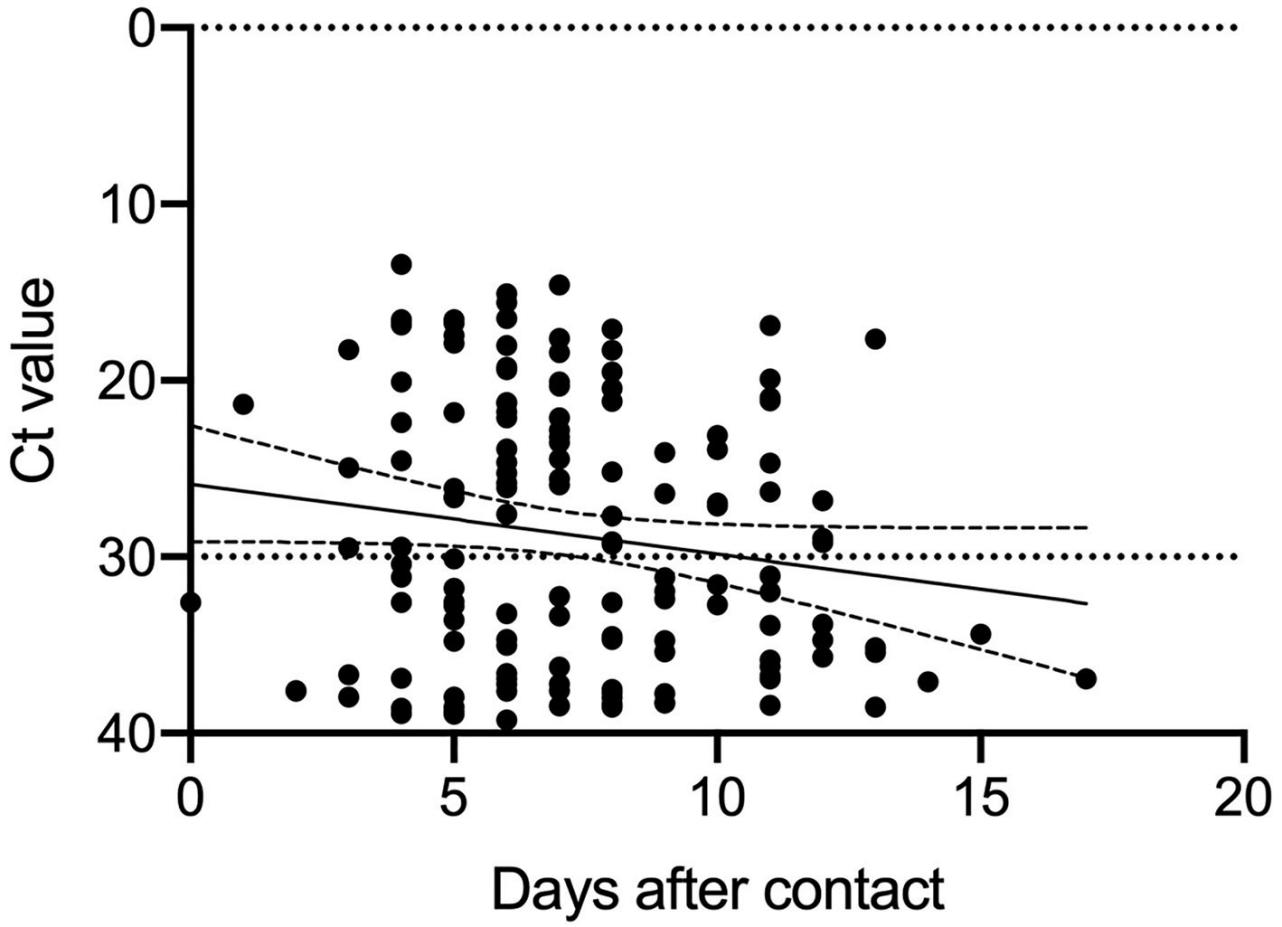
Ct values of the specimens taken from the close contact tracing subjects.

Since positive PCR results alone do not mean that infectious virus is shed, based on previous reports (6), we analyzed the Ct values of the 144 PCR positive samples. The results of the analysis and the duration from specimen collection after close contact are shown in Figure 2. The Ct values were larger (reflecting reduced viral amounts) as time passed after the close contact. We found positive correlation between the Ct value and the days after contact (Ct: y = 0.3979 x + 25.87 [slope: 95% CI, −0.0203–0.8161]) (*p* = 0.062). As the time since specimen collection increased, the Ct-values tended to increase, indicating a decrease in the viral load of the specimens. According to this equation, 11.6–17.5 days were required to achieve a Ct-value ≥30. Figure 1A shows the subjects with a high viral load (Ct ≤ 30) and the days after close contact. PCR positivity could be detected between days 3 and 13 with the highest rate of subjects with a high viral load on day 10 (16.0%).

## Discussion

In this study, we established two major results. First, on the epidemiological close contact tracing, male gender, age ≥ 60, and household contacts were independent infection risk factors for the close contacts of infected persons. Furthermore, symptomatic subjects were considered to be highly suspicious of being infected with SARS-CoV-2. Second, the close contact persons of patients with COVID-19 presented PCR positivity for up to 17 days following the close contact, although subjects with a high viral load (Ct ≤ 30) could be found up to day 13.

PCR positivity was detected in close contacts up to 17 days after the contact. The subjects tested between days 14 and 17 after the close contact were suggested still possessed viral RNA in the saliva or nasopharyngeal specimens but not infective to other persons (Figures 1A,B). No subject exhibited high viral load (Ct-value < 30) on days 14–17. Therefore, they were considered to exhibit a reduced viral load and less infectious to other people. In order to intervene in the second infection from the close contact subjects of patients with COVID-19, a contact tracing test had to be performed between days 3–13 after the close contact to prevent further expansion. This approach was applicable for all the subjects with a close contact history regardless their symptomatic or asymptomatic status at the time of being PCR tested. Saliva specimens were reported to be less sensitive for PCR testing than nasopharyngeal specimens (15). In contrast, another research has reported that saliva specimens were more sensitive for PCR testing in asymptomatic or mild COVID-19 patients (16).

The Ct value was reportedly dependent on the period from infection and useful for determining infectivity (17). That is, as the Ct value reflects the viral load, the subjects with higher Ct-values (Ct ≥ 30) are thought to be potentially less infectious. In particular, the samples with Ct-values over 30 were no longer cultured and did not show infectivity (6, 18). Hence, during the contact tracing, the infectious potential lasts for ~2 weeks after the contact even in the asymptomatic subjects. The period at risk of infectious potential that we established is consistent with the previous estimation of the COVID-19 incubation and elimination periods. Considering the infectious potential, the PCR positive subjects, even the asymptomatic ones, should be properly quarantined to break the transmission chain.

Male gender, age ≥ 60, household contact, and symptomatic subjects were the four independent infection risk factors for the close contacts in this study. It is reasonable to think that symptomatic patients were mostly PCR positive. The gender-based immunity difference could also be considered as an underlying mechanism. About gender differences in COVID-19 epidemiology, a previous study reported that more male than female patients might tend to be severe by the disease (19). Therefore, there are reasons to speculate that men are more susceptible to COVID-19 than women. Among the contact types, household contact was the most common and an independent risk factor for COVID-19. Therefore, the strategy of isolating the infected persons within the household could be reasonable.

According to our study, the potential reason for the low infection numbers in Japan may be effective contact tracing, which successively detected to isolate the symptomatic and asymptomatic COVID-19 patients. At the same time, the manual survey for contact tracing driven by public health centers of local governments has a limit when the infection spreads explosively. The survey at the time of infection spread prone to staff shortages and delays in investigations. An emergency volunteer-run contact tracing survey was reported to fall short of adequate time and information (20). Furthermore, only 3 days delay of isolation of infectious person was reported leading to infection control failure (21). For that reason, digital contact tracing is expected, but ethical issues have been pointed out. Therefore, it is important to inform people of the high-risk situation (male gender, age ≥ 60, household contact, and symptomatic subjects) we have reported and to enable voluntary quarantine for 2 weeks. In that sense, the results of our study contribute to the infection control of SARS-Cov-2.

Nevertheless, our study has certain limitations. First, it consisted of a retrospective review at a single center with a limited number of participants. The characteristics of our patients referred by the public health center might depend on the study region and hospital characteristics. It was difficult for us to continuously pursue the subjects and obtain PCR specimens. The situations such as knowledge and approach to COVID-19 and the number of newly diagnosed COVID-19 patients had changed during this study, this study was possibly heterogenous in the beginning and the end of this study. The number of positive cases may have been underestimated if some subjects became positive at any timepoint after our examination. We were not able to directly compare nasopharyngeal swab specimens and saliva on the same subject. It was a limitation that we were not able to employ nasopharyngeal swab or saliva for PCR test throughout this study. The contact with patients with COVID-19 was self-reported, and the closeness of the contact and infection probability might depend on the situation reported. Therefore, further large-scale epidemiological studies would be required to obtain more concrete evidence on the SARS-CoV-2 transmission dynamics in the tracing of close contacts.

## Data Availability Statement

The raw data supporting the conclusions of this article will be made available by the authors, without undue reservation.

## Ethics Statement

The studies involving human participants were reviewed and approved by the Institutional Review Board of Yokohama City University Hospital (B200200047). Written informed consent from the participants' legal guardian/next of kin was not required to participate in this study in accordance with the national legislation and the institutional requirements.

## Author Contributions

SM and HK contributed to the conception and design of the study, acquisition of data, and analysis and interpretation of data. SM, HK, NT, KS, HO, CK, and SU collected the clinical data. SM wrote the draft. HK conducted the statistical analysis. HN and TY supervised all aspects of this study. All authors revised critically, approved the final version of the manuscript, and attest to meeting the four ICMJE authorship criteria.

## Conflict of Interest

HK reports grants from Shionogi & Company, Limited, during the conduct of the study, outside the submitted work. The remaining authors declare that the research was conducted in the absence of any commercial or financial relationships that could be construed as a potential conflict of interest.

## Publisher's Note

All claims expressed in this article are solely those of the authors and do not necessarily represent those of their affiliated organizations, or those of the publisher, the editors and the reviewers. Any product that may be evaluated in this article, or claim that may be made by its manufacturer, is not guaranteed or endorsed by the publisher.
